# High-Accuracy Three-Dimensional Deformation Measurement System Based on Fringe Projection and Speckle Correlation

**DOI:** 10.3390/s23020680

**Published:** 2023-01-06

**Authors:** Chuang Zhang, Cong Liu, Zhihong Xu

**Affiliations:** School of Science, Nanjing University of Science and Technology, Nanjing 210094, China

**Keywords:** fringe projection profilometry, digital image correlation, fringe and speckle separation, neural network, high accuracy

## Abstract

Fringe projection profilometry (FPP) and digital image correlation (DIC) are widely applied in three-dimensional (3D) measurements. The combination of DIC and FPP can effectively overcome their respective shortcomings. However, the speckle on the surface of an object seriously affects the quality and modulation of fringe images captured by cameras, which will lead to non-negligible errors in the measurement results. In this paper, we propose a fringe image extraction method based on deep learning technology, which transforms speckle-embedded fringe images into speckle-free fringe images. The principle of the proposed method, 3D coordinate calculation, and deformation measurements are introduced. Compared with the traditional 3D-DIC method, the experimental results show that this method is effective and precise.

## 1. Introduction

Optical metrology is widely used in biomedicine, reverse engineering, bridge monitoring and other fields [1,2,3], because of its non-contact, speed and high-accuracy advantages [4,5,6,7]. Fringe projection profilometry (FPP) [8,9] and digital image correlation (DIC) [10,11] are two common non-interference measurement methods.

FPP uses a projector to project fringes onto the measured object [12]. The phase information [13,14] can be solved from the deformed fringe images, and the three-dimensional (3D) shape of the object can be reconstructed by the phase. However, it is only sensitive to the out-of-plane displacement of the measured object in deformation measurements. It cannot achieve the tracking of object points.

DIC employs a speckle texture on the surface of the measured object as the deformation information carrier [15]. Two-dimensional DIC (2D-DIC) [16] adopts a single camera to capture images, making it is easy to operate, but it can only measure in-plane deformation. Three-dimensional DIC (3D-DIC) [17] can measure the 3D deformation of objects with high accuracy, but it requires synchronous triggering of multiple cameras. Furthermore, the filtering effects of subset windows will lower the accuracy of non-uniform deformation fields.

The combination of DIC and FPP can overcome the disadvantages of their respective methods. However, the separation of fringe and speckle images is the key procedure. Therefore, Shi et al. [18] obtained a surface texture image of a measured object from phase-shifting fringe images. Because the grey gradient of the speckle texture on the surface of the object changes little, it is not necessary to separate the fringe images to realize the combination of DIC and FPP for the measurement. However, in practice, in order to improve the accuracy of DIC measurement, it is necessary to spray or transfer the speckle with large grey gradient changes on the measured surface. This also leads to poor quality of the captured fringe images, which cannot be directly used for calculation. This requires us to separate the fringe and speckle images. Felipe Sese et al. [19] used a multisensory camera and laser structural illumination to separate the color encoding of characterized fringe and speckle patterns, and realized the measurement of 3D displacement. However, the measurement errors of this method are large, and this method is not suitable for high-speed measurements.

Therefore, in this paper, a fringe and speckle image separation method based on deep learning technology is proposed, which can significantly improve the modulation of fringe images [20], and the accuracy of 3D deformation measurements. A set of three-step phase-shifting speckle-embedded fringe images is converted into speckle-free fringe images using a convolutional neural network (CNN). This method with high efficiency can automatically produce speckle-free images using the trained CNN model.

## 2. Principle

**Figure 1:**
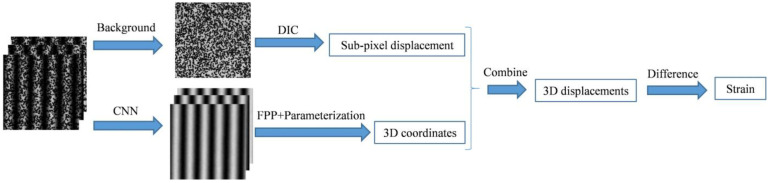
Flow chart of the proposed measurement method.

A flow chart of the proposed measurement method is shown in Figure 1. Firstly, speckle images are extracted from the background light grey intensity of the phase-shifting fringe images. DIC is applied to calculate the sub-pixel displacement of the measured object before and after deformation. At the same time, high-quality fringe images are separated from the speckle-embedded fringe images using CNN. The 3D coordinates of the measured object before and after deformation can be obtained via FPP and the parameterization results of the system. Secondly, the least-squares method is used to solve the integer pixel 3D coordinates before deformation and the 3D coordinates corresponding to the sub-pixel after deformation. The 3D displacement can be achieved by subtracting the corresponding 3D coordinates. At last, the difference method is used to solve the full-field strain. It is necessary to establish local coordinate systems. The 3D coordinates before deformation and 3D displacement are converted to the corresponding local coordinate systems, and the strain tensor can be obtained by fitting the 3D displacement in the local system. The above contents will be introduced in the following four subsections.

### 2.1. Analysis of the Influence of Speckle

The phase-shifting profilometry (PSP) method calculates the phase information of the surface of the measured objects using fringe images. Ideally, a set of sinusoidal fringes are projected by the projector, and the intensity of the image captured by the camera can be expressed as:

(1) $$I_{n}(x,y)=A(x,y)+B(x,y)\cos\left[\varphi (x,y)+\Delta \varphi_{n}\right]$$

where $I_{n}(x,y)\in [0,\;255]$ is the grey intensity of the standard sinusoidal fringe images captured by the camera, A(x,y) is the background grey intensity, B(x,y) is the surface reflectivity, φ(x,y) is the phase to be obtained, which contains the 3D information of the object, $\Delta \varphi_{n}$ is the phase-shifting phase, and n=1,2,3⋯,N represents the number of phase-shifting steps.

The least-squares phase solution method for *N*-step phase-shifting can be expressed as [21]:

(2) $$\varphi (x,y)=\arctan\left[\frac{-\sum_{n=1}^{N}I_{n}\sin(\Delta \varphi_{n})}{\sum_{n=1}^{N}I_{n}\cos(\Delta \varphi_{n})}\right]$$

In order to establish the speckle model of images, a random number of speckles [22] in $I_{n}(x,y)$ are added. Then, the grey intensity of the speckle-embedded fringe images is expressed as:

(3) $${I^{\prime }}_{n}(x,y)=\left\{\begin{array}{cc} I_{n}(x,y) & S_{n}(x,y)=255\\ \;\;\;\;\;0 & S_{n}(x,y)=0 \end{array}\right.$$

where ${I^{\prime }}_{n}(x,y)$ is the grey intensity of speckle-embedded fringe images, and S(x,y) is the random number of speckles.

**Figure 2:**
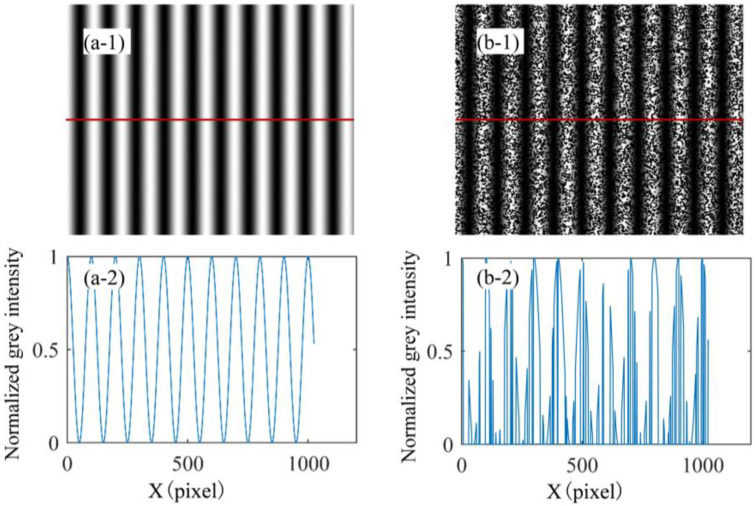
Effect of speckle on fringe image quality. (**a1**,**a2**) The speckle-free fringe image and grey intensity, respectively; (**b1**,**b2**) the speckle-embedded fringe image and grey intensity, respectively.

According to Equations (1) and (3), a speckle-free fringe image with an initial phase value of 0 rad and a speckle-embedded fringe image with an initial phase value of 0 rad, generated via computer simulation, are shown in Figure 2a1 and Figure 2b1, respectively. In the middle row of the image, there is a red line, and the grey intensity distribution of the two cases is shown in Figure 2a2 and Figure 2b2, respectively. The grey intensity distribution of the speckle-free fringe image is a standard sinusoidal curve, while the speckle-embedded fringe image is irregular. If the PSP is applied to solve the phase value of the image directly, as shown in Figure 2b1, it will inevitably induce large phase errors. Therefore, Section 2.2 will describe, in detail, how to extract the fringe pattern based on the deep learning model.

### 2.2. Fringe and Speckle Pattern Separation Based on Deep Learning Model

**Figure 3:**
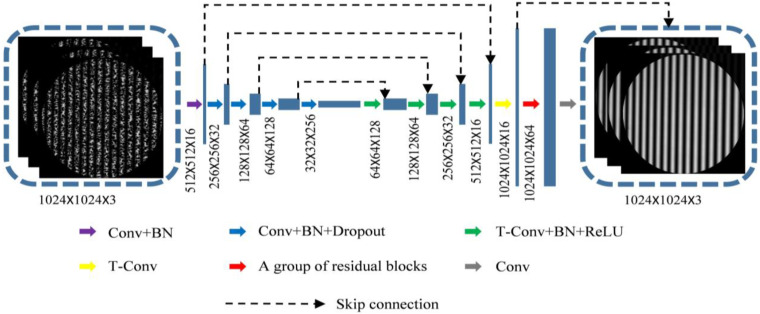
U-shaped CNN structure.

As shown in Figure 3, a U-shaped convolutional neural network (CNN) structure with great feature extraction performance is constructed [23]. Three-step phase-shifting speckle-embedded fringe images are used as the input of the CNN, while speckle-free fringe images are used as the output of the CNN. The U-shaped structure is divided into two parts: feature extraction and feature fusion. The feature extraction part consists of the following operations: convolution (Conv) and batch normalization (BN), and Conv, BN and Dropout by four times. The feature fusion part consists of the following operations: transposed convolution (T-Conv), BN and rectified linear unit (ReLU) by four times; T-Conv; and a group of residual structures and Conv. At the same time, in order to retrieve the missing information, the method of skip connection is used to connect the high-level information and the low-level information to achieve pixel-level information acquisition. The residual block structure includes Conv, residual block and Conv. This structure alleviates the gradient disappearance problem caused by increasing the depth in neural networks.

The loss function of CNN is shown in Equation (4):

(4) $$Loss=\frac{1}{3\times H\times W}\sum_{n=1}^{3\times H\times W}{\left(I_{n}^{out}-I_{n}^{}\right)}^{2}$$

where *H* and *W* are the height and width of the image, respectively, $I_{n}^{out}$ and $I_{n}^{}$ are the output of the network and the given real output.

The adaptive moment estimation (ADAM) learning algorithm is applied in CNN. For parameter selection, the batch size is 2, the starting learning rate is 1 × 10^−3^, and the learning rate will be multiplied by 0.1 after every 200 training rounds.

### 2.3. Three-Dimensional Displacement Calculation

In the combination of DIC and FPP, DIC is mainly utilized to solve the sub-pixel high-accuracy image coordinate displacement of objects before and after deformation. Therefore, the corresponding points before and after deformation can be found. However, since the accuracy of DIC can be reached at around 0.01 pixels, the least-squares curve fitting algorithm is employed to solve the corresponding 3D coordinates of the image sub-pixel coordinates. The first-order polynomial is selected if the measured object with a complex-surface, higher-order polynomial can be employed. The 3D coordinates (*X*, *Y*, *Z*) of the reference subset center and the deformed subset center can be expressed as:

(5) $$\left\{\begin{array}{c} X(x,y)=a_{0}+a_{1}x+a_{2}y\\ Y(x,y)=b_{0}+b_{1}x+b_{2}y\\ Z(x,y)=c_{0}+c_{1}x+c_{2}y \end{array}\right.$$

where (*x*, *y*) denote the local image coordinates of the subset window, and *a*_0_, *a*_1_, *a*_2_, *b*_0_, *b*_1_, *b*_2_, *c*_0_, *c*_1_ and *c*_2_ are the fitting coefficients.

A (2*M* + 1) × (2*M* + 1) subset window area is selected to the nearest integer pixel around the calculation point as the center for fitting.

(6) PS=W

(7) $$P=\left[\begin{array}{ccc} 1 & x_{0}-M & y_{0}-M\\ 1 & x_{0}-M & y_{0}-M+1\\ \cdots & \cdots & \cdots \\ 1 & x_{0}+M & y_{0}+M-1\\ 1 & x_{0}+M & y_{0}+M \end{array}\right]\begin{array}{lll} & & S=\left[\begin{array}{lll} a_{0} & b_{0} & c_{0}\\ a_{1} & b_{1} & c_{1}\\ a_{2} & b_{2} & c_{2} \end{array}\right] \end{array}$$

(8) $$W=\left[\begin{array}{ccc} X(x_{0}-M,y_{0}-M) & Y(x_{0}-M,y_{0}-M) & Z(x_{0}-M,y_{0}-M)\\ X(x_{0}-M,y_{0}-M+1) & Y(x_{0}-M,y_{0}-M+1) & Z(x_{0}-M,y_{0}-M+1)\\ \cdots & \cdots & \cdots \\ X(x_{0}+M,y_{0}+M-1) & Y(x_{0}+M,y_{0}+M-1) & Z(x_{0}+M,y_{0}+M-1)\\ X(x_{0}+M,y_{0}+M) & Y(x_{0}+M,y_{0}+M) & Z(x_{0}+M,y_{0}+M) \end{array}\right]$$

where (*x*_0_, *y*_0_) denote the nearest integral pixel coordinate of (*x*, *y*), **P** is the coordinate matrix of all integral pixels in the selected subset window area, **S** is the fitting coefficient matrix, and **W** is the 3D coordinate matrix corresponding to all integer pixel coordinates in the selected subset window area.

When the fitting coefficients are already calculated, the 3D coordinates of the corresponding reference and deformed object points can be obtained using Equation (5). The 3D displacement (*U*, *V*, *W*) in the global world coordinate system can be expressed as:

(9) $$\left[\begin{array}{c} U\\ V\\ W \end{array}\right]=\left[\begin{array}{c} X_{2}\\ Y_{2}\\ Z_{2} \end{array}\right]-\left[\begin{array}{c} X_{1}\\ Y_{1}\\ Z_{1} \end{array}\right]$$

where (*X*_1_, *Y*_1_, *Z*_1_) and (*X*_2_, *Y*_2_, *Z*_2_) represent the 3D coordinates of the reference subset center and the deformed subset center calculated using Equations (5)–(8), respectively.

### 2.4. Full-Field Strain Measurements

In Section 2.3, the 3D coordinates of the reference subset center are (*X*, *Y*, *Z*), and the 3D displacement (*U*, *V*, *W*) in the global world coordinate system are calculated. In order to calculate the full-field strain of the measured surface, the first step is to establish a local coordinate system through the plane fitting of the local region centered on the point. The local coordinate system takes the vertical fitting plane facing outward as the positive direction of the *Z* axis; the *X* axis and *Y* axis are perpendicular to the *Z* axis, respectively, and can be artificially specified. Then, we calculate the rotation matrix ***R*** and the translation vector ***T*** from the world coordinate system to the local coordinate system. The 3D coordinates before deformation and the 3D displacement are transformed into the corresponding local coordinate systems through the rotation matrix ***R*** and translation vector ***T***.

(10) $$\left[\begin{array}{c} X_{e}\\ Y_{e}\\ Z_{e} \end{array}\right]=R\left[\begin{array}{c} X\\ Y\\ Z \end{array}\right]+T$$

(11) $$\left[\begin{array}{c} U_{e}\\ V_{e}\\ W_{e} \end{array}\right]=R\left[\begin{array}{c} U\\ V\\ W \end{array}\right]+T$$

where $\left(X_{e},Y_{e},Z_{e}\right)$ are the 3D coordinates in the local coordinate system, and $\left(U_{e},V_{e},W_{e}\right)$ are the 3D displacement in the local coordinate system.

The displacement field function is obtained via quadric surface fitting, and the strain tensor can be calculated using the field functions. The fitting forms of the field function are as follows:

(12) $$\left\{\begin{array}{l} U_{e}=a_{1}^{X}X_{e}^{2}+a_{2}^{X}Y_{e}^{2}+a_{3}^{X}X_{e}Y_{e}+a_{4}^{X}X_{e}+a_{5}^{X}Y_{e}+a_{6}^{X}\\ V_{e}=a_{1}^{Y}X_{e}^{2}+a_{2}^{Y}Y_{e}^{2}+a_{3}^{Y}X_{e}Y_{e}+a_{4}^{Y}X_{e}+a_{5}^{Y}Y_{e}+a_{6}^{Y}\\ W_{e}=a_{1}^{Z}X_{e}^{2}+a_{2}^{Z}Y_{e}^{2}+a_{3}^{Z}X_{e}Y_{e}+a_{4}^{Z}X_{e}+a_{5}^{Z}Y_{e}+a_{6}^{Z} \end{array}\right.$$

where $\left(a_{1}^{X},a_{2}^{X},a_{3}^{X},a_{4}^{X},a_{5}^{X},a_{6}^{X}),(a_{1}^{Y},a_{2}^{Y},a_{3}^{Y},a_{4}^{Y},a_{5}^{Y},a_{6}^{Y}\right)$ and $\left(a_{1}^{Z},a_{2}^{Z},a_{3}^{Z},a_{4}^{Z},a_{5}^{Z},a_{6}^{Z}\right)$ are the coefficients of each field function. Then, the Lagrange strain tensor parameters are calculated based on following equations.

(13) $$\left\{\begin{array}{l} \varepsilon_{xx}=\frac{\partial U_{e}}{\partial X_{e}}+\frac{1}{2}\left[{\left(\frac{\partial U_{e}}{\partial X_{e}}\right)}^{2}+{\left(\frac{\partial V_{e}}{\partial X_{e}}\right)}^{2}+{\left(\frac{\partial W_{e}}{\partial X_{e}}\right)}^{2}\right]\\ \varepsilon_{yy}=\frac{\partial V_{e}}{\partial Y_{e}}+\frac{1}{2}\left[{\left(\frac{\partial U_{e}}{\partial Y_{e}}\right)}^{2}+{\left(\frac{\partial V_{e}}{\partial Y_{e}}\right)}^{2}+{\left(\frac{\partial W_{e}}{\partial Y_{e}}\right)}^{2}\right]\\ \varepsilon_{zz}=\frac{\partial W_{e}}{\partial Z_{e}}+\frac{1}{2}\left[{\left(\frac{\partial U_{e}}{\partial Z_{e}}\right)}^{2}+{\left(\frac{\partial V_{e}}{\partial Z_{e}}\right)}^{2}+{\left(\frac{\partial W_{e}}{\partial Z_{e}}\right)}^{2}\right]\\ \varepsilon_{xy}=\frac{1}{2}\left(\frac{\partial U_{e}}{\partial Y_{e}}+\frac{\partial V_{e}}{\partial X_{e}}\right)+\frac{1}{2}\left[\left(\frac{\partial U_{e}}{\partial X_{e}}\frac{\partial U_{e}}{\partial Y_{e}}\right)+\left(\frac{\partial V_{e}}{\partial X_{e}}\frac{\partial V_{e}}{\partial Y_{e}}\right)+\left(\frac{\partial W_{e}}{\partial X_{e}}\frac{\partial W_{e}}{\partial Y_{e}}\right)\right]\\ \varepsilon_{yz}=\frac{1}{2}\left(\frac{\partial V_{e}}{\partial Z_{e}}+\frac{\partial W_{e}}{\partial Y_{e}}\right)+\frac{1}{2}\left[\left(\frac{\partial U_{e}}{\partial Y_{e}}\frac{\partial U_{e}}{\partial Z_{e}}\right)+\left(\frac{\partial V_{e}}{\partial Y_{e}}\frac{\partial V_{e}}{\partial Z_{e}}\right)+\left(\frac{\partial W_{e}}{\partial Y_{e}}\frac{\partial W_{e}}{\partial Z_{e}}\right)\right]\\ \varepsilon_{zx}=\frac{1}{2}\left(\frac{\partial W_{e}}{\partial X_{e}}+\frac{\partial U_{e}}{\partial Z_{e}}\right)+\frac{1}{2}\left[\left(\frac{\partial U_{e}}{\partial Z_{e}}\frac{\partial U_{e}}{\partial X_{e}}\right)+\left(\frac{\partial V_{e}}{\partial Z_{e}}\frac{\partial V_{e}}{\partial X_{e}}\right)+\left(\frac{\partial W_{e}}{\partial Z_{e}}\frac{\partial W_{e}}{\partial X_{e}}\right)\right] \end{array}\right.$$

## 3. Experiments and Results

In order to verify the effectiveness of the proposed method, three sets of experiments were conducted. They are fringe image extraction, 3D displacement and full-field strain measurements.

### 3.1. Fringe Image Extraction

**Figure 4:**
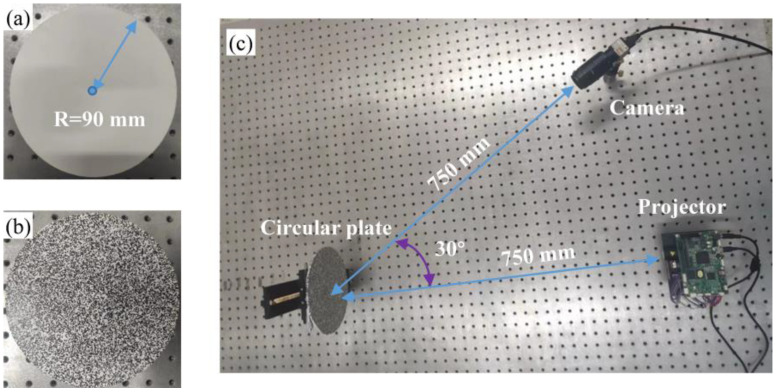
The experimental setup. (**a**) Circular plate without speckles; (**b**) circular plate with speckles; (**c**) the experimental setup.

In Figure 4, the experimental system includes a DLP projector (LightCrafter 4500, Manufacturer Texas Instruments, Headquartered in Dallas, TX, USA) with a resolution of 912 × 1140 pixels^2^, and an IDS UI-3370CP (Manufacturer IDS, Headquartered in Obersulm, Germany) camera with a resolution of 2048 × 2048 pixels^2^ and that operates at 80 frame rates per second. A customized acrylic circular plate is employed as the measured object in the experiment, with a radius of 90 mm. The distances from the camera and projector to the circular plate are about 750 mm, and the angle between the camera and the projector is about 30°.

In order to simulate the real situation, real experimental images are captured by the camera as the training and testing datasets of the CNN. The projector projects a set of three-step phase-shifting speckle-embedded fringe images and a set of three-step phase-shifting speckle-free fringe images to the circular plate, respectively. Two-hundred and forty training image datasets are captured by adjusting the projected speckle size. Next, speckles are generated using Equation (3), and the speckles are transferred to the circular plate via water transfer, as shown in Figure 4b. The projector projects a set of three-step phase-shifting speckle-free fringe images, and the camera captures them as testing datasets. GPU (NVIDIA GeForce RTX 2080TI, 32 GB of RAM, Manufacturer NVIDIA, Headquartered in Santa Clara, CA, USA) and CPU (Intel Xeon Platinum, 96 GB of RAM, Manufacturer intel, Headquartered in Santa Clara, CA, USA) multithreading techniques are employed to accelerate the process of training.

**Figure 5:**
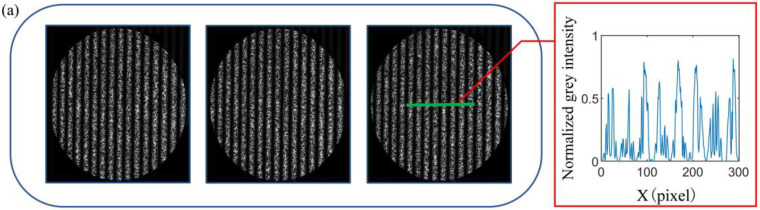

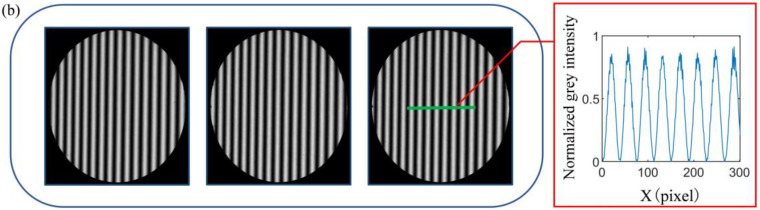
Experimental results. (**a**) The input speckle-embedded fringe images of CNN and grey intensity; (**b**) the output speckle-free fringe images of CNN and grey intensity.

In Figure 5, (a) shows the input fringe images of CNN and grey intensity at the green line, and (b) shows the fringe images predicted by CNN and grey intensity at the green line. It is obvious that CNN can effectively separate fringe images and improve the quality of the fringe images.

### 3.2. Three-Dimensional Displacement Measurements

The circular plate is rotated about 5 degrees, and speckle-embedded fringe images before and after rotation are captured. The fringe images can be extracted by the CNN. The phase value of the circular plate before and after deformation can be calculated using the 3-step phase-shifting algorithm. The parameters of the experimental system can be calibrated using the method proposed by Zhang [24], and the coordinates of the *X*, *Y* and *Z* directions of the circular plate in the global world coordinate system can be obtained.

**Figure 6:**
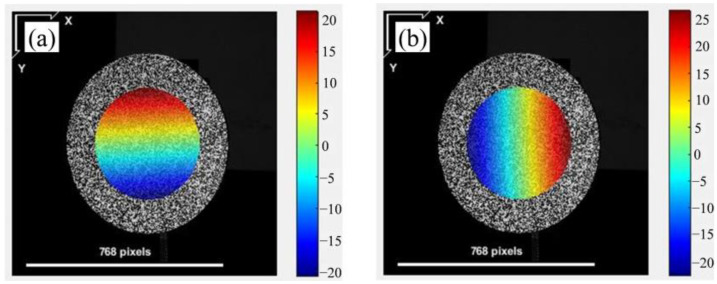
The displacement of integer pixel position. (**a**) Displacement in *x* direction; (**b**) displacement in *y* direction.

Speckle images can be obtained from the background grey intensity of the phase-shifting speckle-embedded fringe images. The circular area in the middle of the circular plate is selected as the calculation area. As shown in Figure 6, (a) shows *x*-direction displacement of the circular plate along the *y* direction in the image, and (b) shows *y*-direction displacement of the circular plate along the *x* direction in the image. Their units are pixels. 

**Figure 7:**
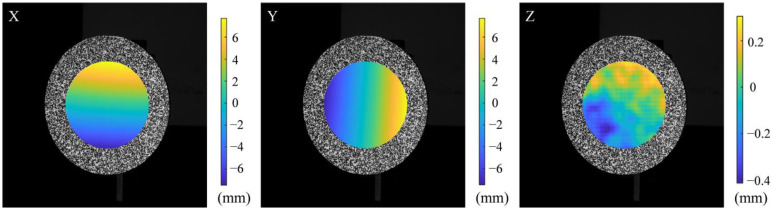
Full-field displacement in the *X*, *Y* and *Z* directions.

The corresponding relationship before and after rotation can be found using the DIC algorithm according to Section 2.3. The fitting subset window area is 29 × 29 pixels^2^. According to Equations (5)–(8), polynomial fitting is performed to find the corresponding 3D coordinate relationship before and after rotation. The displacement in the three directions can be obtained by subtracting the 3D coordinates of the corresponding points. The full-field displacement in the three directions are shown in Figure 7.

The total displacement of the circular plate can be expressed as:

(14) $$D=\sqrt{D_{x}^{2}+D_{y}^{2}+D_{z}^{2}}$$

where *D* is the total displacement, and *D_x_*, *D_y_* and *D_z_* denote the displacement in the *X*, *Y* and *Z* directions, respectively.

**Figure 8:**
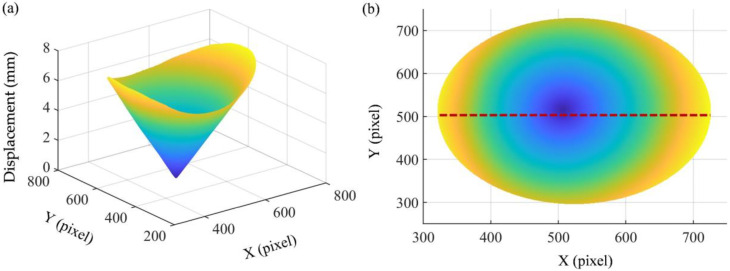
The total displacement. (**a**) The stereogram of the full-field displacement; (**b**) the planar graph of the full-field displacement.

**Figure 9:**
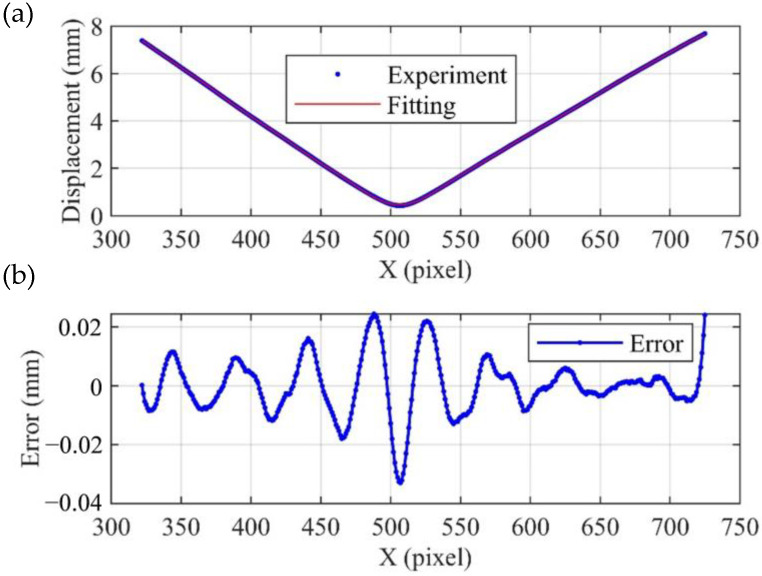
The displacement at the dashed line. (**a**) Comparison of experimental results and fitting results; (**b**) error.

Figure 8a shows a stereogram of the full-field displacement, and Figure 8b shows a planar graph of the full-field displacement. The full-field displacement is funnel-shaped with nearly zero displacement in the middle and large displacement in the periphery. The displacement increases linearly along the radial direction. Figure 9a shows the total displacement on the red dashed line. Accurate displacement can be obtained via Fourier curve fitting. Figure 9b shows the error between total displacement and fitting displacement. It can be seen that the error values are mainly distributed in the range of ±0.02 mm, with only a few exceeding this range. In this experiment, the circular plate takes up about half of the camera’s field of view. The measurement error is only about 0.006%. 

### 3.3. Full-Field Strain Measurements

**Figure 10:**
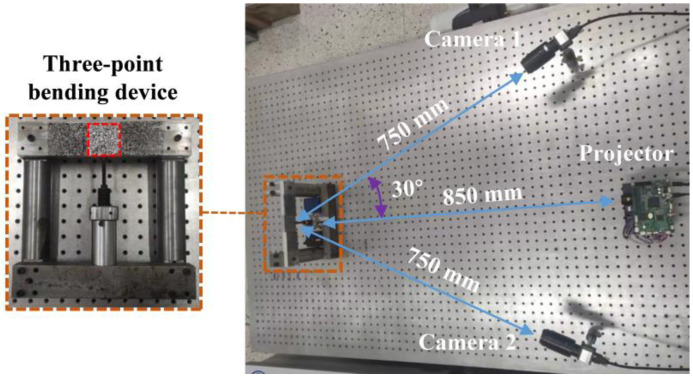
Experimental setup.

Strain reflects the relative deformation of an object under stress. It is an essential physical quantity used to measure the mechanical properties of objects. As shown in Figure 10, camera 1 and the projector form FPP combined with a DIC measurement system. Camera 1 and camera 2 constitute a 3D-DIC measurement system. They are placed symmetrically about the projector. The distance from camera 1 to the three-point bending specimen is about 750 mm. The distance from the projector to the three-point bending specimen is about 850 mm. The angle between camera 1 and the projector is about 30°. Two methods are used to measure the strain in the red dotted area of the three-point bending specimen.

**Figure 11:**
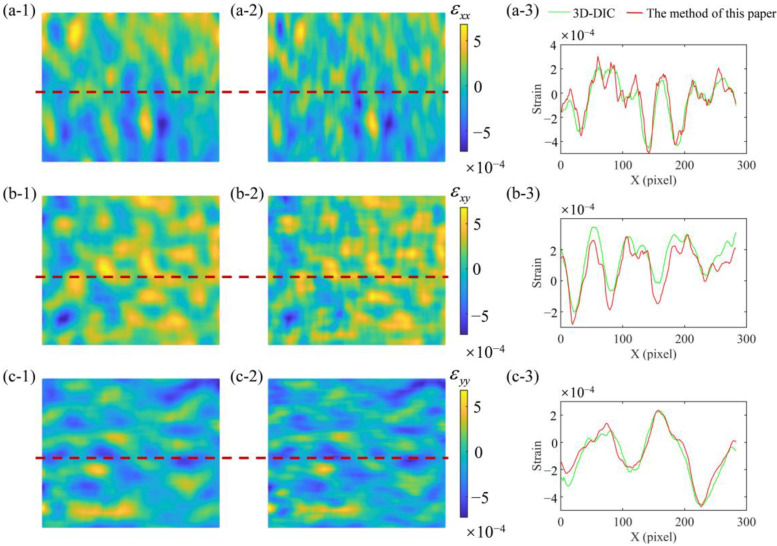
The distribution of full-field strain. (**a1**,**b1**,**c1**) the full-field strain distributions calculated using 3D-DIC; (**a2**,**b2**,**c2**) the full-field strain distributions calculated using the method of this paper; (**a3**,**b3**,**c3**) the strain solved using two methods at the red dotted line.

Since it is impossible to know the changes of the surface below the measured surface, only *Ԑ*_xx_, *Ԑ*_xy_ and *Ԑ*_yy_ can be calculated. Figure 11a1,b1,c1 are the full-field strain distribution calculated using 3D-DIC, Figure 11a2,b2,c2 are the full-field strain distributions calculated using the method of this paper, and Figure 11a3,b3,c3 are the strain solved using two methods at the red dotted line. By comparison, the distribution of full-field strain calculated in this paper is basically the same as that calculated using 3D-DIC. The strain difference between the two methods is only 1 × 10^−4^. Therefore, this method can measure accurate strain results.

## 4. Discussion of Measurement Results under Different Exposure Times

Next, the applicability of the proposed measurement method in high- and low-exposure experimental scenes is discussed.

**Figure 12:**
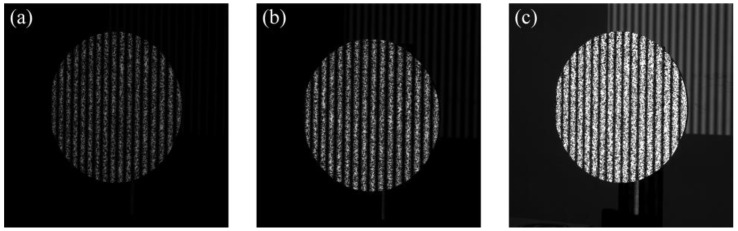
Images acquired at different exposure times. (**a**) Exposure time of 20 ms; (**b**) exposure time of 40 ms; (**c**) exposure time of 80 ms.

In the experiment of this paper, when the camera exposure time is 40 ms, the gray intensity of the image is in a reasonable range. The camera exposure time was adjusted to 20 ms for the low-exposure experimental scene, and the camera exposure time was adjusted to 80 ms for the high-exposure experimental scene. The experimental images collected under different exposure times are shown in Figure 12.

**Figure 13:**
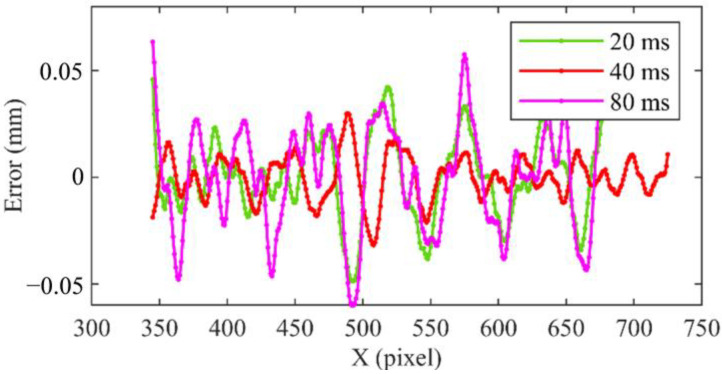
Comparison of displacement measurement error under different exposure times.

Figure 13 shows an error comparison of 3D displacement of a line under the exposure times of 20 ms, 40 ms and 80 ms. It can be clearly seen that the error reaches ±0.05 mm with high and low exposure. In Table 1, the RMSE of displacement measured with low exposure time (20 ms) is 0.02 mm, the RMSE measured with normal exposure (40 ms) is 0.01 mm, and the RMSE measured with high exposure (80 ms) is 0.03 mm. Therefore, the method presented in this paper can be used to measure both high- and low-exposure experimental scenes and has high accuracy.

## 5. Conclusions

In this paper, a high-accuracy 3D deformation measurement system based on fringe projection and speckle correlation is proposed, which can effectively eliminate the influence of speckles on fringe image quality. The difficulty of combining DIC with FPP is solved. The method has high accuracy in displacement measurement and can be effectively applied to high- and low-exposure experimental scenes. A similar trend to 3D-DIC can be obtained in strain measurements.

Before each measurement, the measurement system in this paper needs to be calibrated, and the high-accuracy calibration plate should be selected as far as possible. In addition, the measurement system in this paper can also be calibrated by measuring the standard specimen or the standard deformation.

## Figures and Tables

**Table 1:** Root mean square error of displacement measurement (mm).

Exposure Time	20 ms	40 ms	80 ms
RMSE	0.02	0.01	0.03

## Data Availability

Not applicable.
